# In Silico Analysis of Bioactive Peptides Produced from Underutilized Sea Cucumber By-Products—A Bioinformatics Approach

**DOI:** 10.3390/md20100610

**Published:** 2022-09-28

**Authors:** Tharindu R. L. Senadheera, Abul Hossain, Deepika Dave, Fereidoon Shahidi

**Affiliations:** 1 Department of Biochemistry, Memorial University of Newfoundland, St. John’s, NL A1C 5S7, Canada; 2 Marine Bioprocessing Facility, Centre of Aquaculture and Seafood Development, Marine Institute, Memorial University, St. John’s, NL A1C 5R3, Canada

**Keywords:** sea cucumber by-products, bioinformatics, bioactive peptides, value addition

## Abstract

Bioinformatic tools are widely used in predicting potent bioactive peptides from food derived materials. This study was focused on utilizing sea cucumber processing by-products for generating antioxidant and ACE inhibitory peptides by application of a range of in silico techniques. Identified peptides using LC−MS/MS were virtually screened by PepRank technique followed by in silico proteolysis simulation with representative digestive enzymes using BIOPEP-UWM^TM^ data base tool. The resultant peptides after simulated digestion were evaluated for their toxicity using ToxinPred software. All digestive resistance peptides were found to be non-toxic and displayed favorable functional properties indicating their potential for use in a wide range of food applications, including hydrophobic and hydrophilic systems. Identified peptides were further assessed for their medicinal characteristics by employing SwissADME web-based application. Our findings provide an insight on potential use of undervalued sea cucumber processing discards for functional food product development and natural pharmaceutical ingredients attributed to the oral drug discovery process.

## 1. Introduction

Identification of bioactive peptides has evolved over the years from tedious time-consuming conventional processing steps of purification and characterization into the use of bioinformatic tools to discover the potential precursors of bioactive peptides [1,2]. The use of protein databases and computer-based tools to assess food protein sequences has become an effective and feasible method to discover and identify the bioactive peptides [2]. The bioinformatics approach combines biological mass data using computer science, biology, mathematics and statistical analysis methods [3]. These in silico approaches include diversified tools as databases of protein and peptide sequences, web-based applications for predicting the bioactivities and physicochemical properties of proteins and peptides, identifying structure-functional relationships of peptide as well as programs that enable the theoretical hydrolysis of proteins. The latter is done by calculating quantitative descriptors and recommending the use of appropriate enzymes as endopeptidases or exopeptidases to produce potent peptides from native proteins [4]. Furthermore, use of ‘-omics’ techniques could replace the existing ‘trial and error approach’ in peptide purification and activity research. The downstream purification process of bioactive peptides can be accelerated by the integration of in silico methods with ‘-omics’ approach. The bioinformatic tools, including universal protein and peptide data bases (UniProtKB, SwissProt, BIOPEP and PepBank), are employed to obtain high throughput related to in silico protein digestion and peptide prediction information on potent bioactive peptide sequences [1,2]. Therefore, use of bioinformatics is recognized as an effective mining tool to accelerate the process of discovering bioactive peptides encrypted in different types of protein precursors.

In recent years, much attention has been paid to identifying bioactive peptides originating or produced from seafood processing waste [5,6,7]. It is also identified as an effective bio-refinery approach to address the adverse environmental conditions associated with common waste disposal methods [5,6]. Numerous studies have been conducted for utilizing various marine organisms and their by-products as rich sources of bioactive peptides and essential amino acids [6,7,8]. However, when compared to other marine fisheries, sea cucumbers remain a largely untapped marine protein resource [9,10,11]. In particular, Atlantic Sea cucumber *Cucumaria frondosa* is still considered as an emerging fishery in North Atlantic provinces [9,10]. It is mainly fished for its internal muscle bands and body wall [10,12]. All the other remaining tissues including aquapharyngeal bulb (flower) and internal organs such as digestive tract, gonads, and respiratory tree are discarded as processing waste [11,12,13]. A limited number of studies have reported the potential of utilizing the Atlantic Sea cucumber by-products as precursor materials for bioactive peptides [14,15]. To the best of our knowledge in silico analysis on hydrolysates prepared from sea cucumber by-products have not been studied to assess their potential as inhibitors of angiotensin converting enzyme (ACE). Furthermore, there are no reports on exploring drug likeliness of sea cucumber derived bioactive peptides.

For the first time, the present study reports the application of in silico tools to comprehensively study peptidomic data of protein hydrolysates prepared from Atlantic Sea cucumber by-products as sources of antioxidant and ACE inhibitory peptides. In addition, the aim of the study was also to predict the drug likeliness of sea cucumber derived multifunctional peptides.

## 2. Results and Discussion

### 2.1. Prediction of Bioactive Potential of Identified Peptides from Protein Hydrolysates Samples

Identified peptides from LC-MS/MS analysis were analyzed using “Peptide Ranker (PepRank)” web-based application. PepRank is an in silico tool that predicts the probability of a peptide to be bioactive by ranking them within the range between 0 and 1 [16]. Computational predictions of bioactivity in PepRank generally cover the different classes of bioactive peptides. In addition, it considers the amino acid composition and impact of extracellular status on predictions [17]. Upon submitting the list of identified peptide sequences from each sample, PepRank predicts the probability of the bioactivity of each submitted peptide. The general threshold value is referred as 0.5 and peptides that possess scores above 0.5 are considered bioactive [18]. It is also noteworthy that false positive rates decrease with increasing the threshold values. For example, a false-positive rate of 16% of short peptides at 0.5 threshold will decrease to 6% by increasing the threshold to 0.8. For long chain peptides this will change from 11% to 6% [17]. Furthermore, it may also affect the true-positive rate. Therefore, it is always prudent to select the threshold value carefully based on the purpose of the analysis, i.e., identification of all the true-positives or screening out or reduce the number of false-positives [17,18]. For our study, we selected 0.9 as the threshold value based on suggestion that the probability of a peptide to be bioactive is significantly high when the predicted score is close to 1 [16]. As shown in Table 1, 8 peptides were over 0.9 threshold score in flower whereas 6 peptides were identified from internal organs samples. Among all the predicted values, flower and internal organs shared the same peptide sequence (GPPGPQWPLDF with 0.96) for the highest predicted bioactivity. The predicted bioactive peptides were then analyzed for their specific bioactive potentials with emphasis on antioxidant and ACE inhibitory activities using BIOPEP data base.

### 2.2. In Silico Predictions of Potential Antioxidative Peptides

BIOPEP-UWM^TM^ data base tool was used to identify the antioxidant potential of highest-ranking peptides. Peptide sequences were analyzed based on the profiles of potential bioactive peptides reported in the literature and databases as well as the frequency of occurrence of fragments with a given activity [19]. Identified peptide sequences were submitted to the BIOPEP as query sequences and selected the “antioxidant activity” as bioactivity of interest. Table 2 and Table 3 represent the in silico predictions of antioxidant potential of amino acid sequences found in flower and internal organs, respectively.

According to the BIOPEP-UWM^TM^ data base, 5 out of 8 peptides from flower and 2 out of 6 peptides from internal organs were identified as potential antioxidative peptides. Interestingly, all the three body parts of the sea cucumber shared similar bioactive sequences, namely GPP, WPL and MM. In general, amino acid residues including G (glycine), P (proline), W (tryptophan), L (leucine) and M (methionine) are recognized as some of the most established hydrophobic amino acids associated with antioxidant activity [5,7,20]. For example, tryptophan is an electron-dense aromatic residue that can contribute to the chelation of prooxidant metal ions. Moreover, the indole side chain of tryptophan can scavenge free radicals by electron donation [21]. Similar findings were reported by Zhang et al. [15] in Alcalase-produced peptide fractions from Atlantic Sea cucumber samples. Furthermore, the authors indicated that antioxidant potential of peptides produced using Alcalase was higher than that of trypsin-produced peptides due to the presence of the above-mentioned amino acid residues in their sequences [15]. In addition, the common amino acid sequence found in the sea cucumber body parts, GPPGPQWPLDF, contained phenylalanine (F). Phenylalanine can also act as a hydrogen donor to scavenge free radicals [22]. Presence of hydrophobic and aromatic amino acids is considered as key attributes to the antioxidative property of bioactive peptides [23]. Several antioxidant peptides identified from marine invertebrates including shrimp, jelly fish confer that peptides having most hydrophobic amino acids in their sequences possess strong antioxidant activities, including radical scavenging, reducing power, and inhibition of lipid peroxidation [24] GP (glycine-proline) sequence and particularly the occurrence of proline (P) in high proportion in the entire peptide enhanced its radical scavenging ability, in addition to the direct contribution of tryptophan [25]. When considering lipid peroxidation, it is presumed that increased lipid solubility of peptides due to the presence of hydrophobic amino acids can facilitate the interaction with radical species [22,24]. Therefore, the current findings support the fact that sea cucumber-derived peptides may have the potential to inhibit the lipid peroxidation process. In addition, the synergism of amino acid residues present in the sequence may also contribute to antioxidant properties [24,25,26]. Therefore, the occurrence of potential bioactive sequences inside the identified peptides may enhance opportunities to identify novel antioxidant sources.

### 2.3. In Silico Predictions of Potential ACE Inhibitory Peptides

Computer simulation for the identification of ACE inhibitory activities was conducted using BIOPEP-UWM^TM^ data base tool by selecting the “ACE inhibitory activity” as bioactivity of interest. In silico approaches use chemometrics and information related to sequence homology for understanding and predicting the bioactivity of amino acid sequences submitted to the data base as “query sequence” [27]. According to the findings given in Table 4 and Table 5, all selected bioactive peptides in flower and internal organs possess ACE inhibitory active sequences.

It is evident that all active fragments are either dipeptides or tripeptides. Presence of hydrophobic amino acid residues is the crucial structural feature for ACE inhibitory potential [28]. Hydrophobic amino acids including P (proline), M (methionine), L (leucine), I (isoleucine), F (phenylalanine), and A (alanine) were identified in the predicted potent amino acid sequences. In addition, positioning of hydrophobic amino acids or branched chain amino acid residues at the C-terminal of the sequence and N-terminal aliphatic amino acids are some of the major structural features which favors the ACE binding function [29]. These characteristic features were exhibited in predicted sequences of flower and internal organs. The highest number of potential ACE inhibitory dipeptides, and tripeptides (17) were found in sequence GPPGASGPLGIAGSM from flower. Furthermore, all the other predicted ACE inhibitory peptides shared similar structural features, including the presence of hydrophobic amino acid residues in the sequence. In addition, occurrence of consecutive proline-proline dipeptide in the sequence is markedly effective for antihypertensive activities [7]. The effect of proline towards the ACE inhibitory activity associates with its imidazole ring that exhibits strong interaction with the amino acid residues at the active centers of ACE [30]. According to the most recent studies, more than 50% of the identified ACE inhibitory peptides from gelatin hydrolysates had proline residues in the C-terminal position whereas more than 60% had one proline residue in one of the three C-terminal positions [29,30]. Most of the identified peptides in the present study also had this GP sequence at their C-terminal. Almost all predicted ACE inhibitory peptides in sea cucumber protein hydrolysates were found to have the proline residue in each of their sequences. This further confirms the potential of peptides derived from the North Atlantic Sea cucumber towards ACE inhibition.

However, it has been suggested that ACE inhibitory property of protein hydrolysates could be considered as a collective effect from various peptide chains rather than from a single bioactive peptide [7,29,31]. In addition, it is always recommended to investigate the potential resistance to gastric enzymes of these identified bioactive peptides, once they are expected to exert a beneficial physiological effect after digestion. Thus, all predicted peptides from sea cucumber protein hydrolysates were subjected to in silico simulated gastrointestinal digestion to determine their efficacy upon digestion.

### 2.4. In Silico Simulated Gastrointestinal (GI) Digestion of Bioactive Peptides

Simulated GI digestion that mimics protein degradation in the stomach and small intestine is considered as a rapid and effective tool to determine the stability of peptides against GI proteases [30]. In this regard, in silico simulated GI digestion is an effective approach to investigate the bioactive potential of any peptide of interest prior to assessing their bioavailability and bioaccessibility in vivo. Thus, selected peptides were subjected to in silico proteolysis simulation with representative digestive enzymes; pepsin (EC 3.4.23.1), trypsin (EC 3.4.21.4) and chymotrypsin (EC 3.4.21.1) using “BIOPEP Enzyme(s) Action” program [19]. The current study predicted the potential of peptides derived from sea cucumber protein hydrolysates as the precursors of peptides with antioxidative or ACE inhibitory properties. Table 6 and Table 7 represent the bioactive potential upon GI digestion of peptides derived from flower and internal organs, respectively.

According to the predictions, none of the peptides showed antioxidative potential after the simulated digestion process. This may be due to the scarcity of information in databases related to the potent antioxidative active peptides. It has been suggested that bioinformatics strongly depend on the information included in the data base [1]. However, prediction of releasing ACE inhibitory peptides was found to be similar in all samples. Three peptides (GPPGPQWPLDF, APDMAFPR and GPGMMGP) from flower and internal organs were predicted to release potent ACE inhibitory fragments from their original sequences. Furthermore, frequency of releasing potent ACE inhibitory peptides from each identified peptide was varied as some peptides have more than one active fragment embedded in their sequences. For example, GPPGPQWPLDF sequence was predicted to release two active ACE inhibitory sequences, namely PL and DF, whereas APDMAFPR has the potential to release PR and AF following simulated GI digestion. The release of bioactive peptides was predicted after using the “enzyme(s) action” option of BIOPEP data base (Table 8).

Calculated quantitative parameters of proteolysis include theoretical degree of hydrolysis (DH_t_), the frequency of the release of fragments with a given activity by selected enzymes (A_E_), and the relative frequency of the release of fragments with a given activity by selected enzymes (W). The DH_t_ values for peptides varied from 11.11 to 33.33%. Among them, GPGMMGP showed the highest efficiency (33.33%) on releasing the bioactive enzymes and GPPGPGNAF was the lowest (11.11%). Descriptors like A_E_ and W directly analyzed the potential bioactivity of sea cucumber derived peptides. The higher A_E_ values suggest the possibility of having higher number of peptides with specific activity. The highest A_E_ value (0.25) was observed in APDMAFPR from flower and internal organs [32]. The other remaining peptide sequences, namely GPPGPQWPLDF and GPGMMGP, were common for all samples and their calculated A_E_ values were 0.18 and 0.14, respectively. The highest relative frequency of the release of fragments with a given activity by selected enzymes (W) was 0.33 in APDMAFPR, followed by GPPGPQWPLDF (0.25). Similar W value (0.14) was observed for GPGMMGP and GPPGPGNAF. It was suggested that W values are considered complimentary to A_E_ values [19]. In addition, W values can be correlated with catalytic specificities and the number of recognition sites in each enzyme [33]. Most of the bioactive fragments remaining after in silico simulated digestion of sea cucumber derived peptides possess either P (proline) or phenylalanine (F) in their dipeptide sequences. The resultant bioactive motifs contain PL (proline-leucine), DF (aspartic acid-phenylalanine), GP (glycine-proline), AF (alanine-phenylalanine) and PR (proline-arginine). Similar findings were reported by Iwaniak et al. [32] in their in silico study of identifying biopeptides from collagen derived from various sources including cow, pig, sheep, chicken, duck, horse, salmon, rainbow trout, goat, rabbit and turkey. Authors reported that all the identified motifs which exhibited ACE inhibitory activity contained proline, phenylalanine, glycine, leucine and arginine. As described earlier, previous studies on ACE inhibitory peptides also revealed that proline residues appear to provide resistance to digestive enzymes. The dipeptide sequences with a C-terminal proline and hydroxyproline were reported to be more bioavailable compared to other amino acid sequences [34]. Specifically, the presence of proline is well documented for its distinct ability for binding to ACE and interestingly, most commercially existing inhibitors bear the proline residue in their sequences [35]. Furthermore, some studies have identified that the presence of hydrophobic amino acids at the penultimate position of C-terminus as being a favorable feature for ACE inhibitory potency [36]. Thus, in silico prediction of generating potent ACE inhibitory peptides from sea cucumber samples may contribute to a better understanding of peptide efficiency before performing in vitro and in vivo experiments. Nevertheless, these findings can be used as a preliminary information on the stability of the sea cucumber derived peptides as well as to help determining the relationship between amino acid composition of peptides and their GI digestion.

### 2.5. Prediction of Toxicity and Physicochemical Properties of Released Bioactive Peptide Fractions after In Silico Digestion

In silico proteolysis does not guarantee that the resultant peptides with bioactivities are always safe for use in food and pharmaceutical products. In addition, there is a possibility of identifying novel peptides. Therefore, toxicity evaluation has been suggested as a mandatory approach prior to further development of identified motifs [33]. The software ToxinPred was used for predicting the toxicity of peptides as well as their physicochemical properties including hydrophobicity, hydrophilicity, charge, isoelectric point and molecular weight of the released dipeptides. The toxicity of peptides was investigated based on hybrid model of dipeptide composition and motif scanning [37]. According to the model developed using the machine-learning techniques support vector machine (SVM) based on amino acid compositional analysis, residue preferences combined with quantitative matrix-based and motif-based predictions, ToxinPred creates the platform to assess realistic toxicity of peptides [38]. The developed method reveled that valine (V), threonine (T), arginine (R), glutamine (E), methionine (M), leucine (L), lysine (K), isoleucine (I), phenylalanine (F) and alanine (A) were abundant in non-toxic peptides, whereas cysteine (C), histidine (H) and asparagine (N) were predominant in toxic peptides [38]. Predictions obtained following toxicity analysis show that none of the dipeptides (PL, GF, GP, DF, PR) released from sea cucumber-derived peptide simulated digestion have any potential toxicity. Thus, these findings indicate that sea cucumber-derived peptides can be used as functional food ingredients or nutraceutical products. According to the predictions of ToxinPred, molecular weight of the peptides ranged from 172.20 to 280.29 Da. Out of the five identified ACE inhibitory potent dipeptides obtained from sea cucumber protein hydrolysates, three (PL, GP and AF) had an isoelectric point (pI) of 5.88 with 0 net charge (Table 9).

The remaining peptides, DF and PR, had negative charge (−1) and positive charge (+1) with a pI of 3.88 and 10.11, respectively. Structural properties, including charge, peptide sequence and low molecular size have been identified as influential characteristics for the bioavailability of food-derived peptides [39]. It has been reported that short chain peptides produced from gelatin hydrolysates have the potential to enter blood stream by crossing the intestinal barriers of humans and exert their bioactivity [40]. Generally, small peptide sequences are less prone to further degradation and possess high bioavailability [41]. In addition, most of the reported potent ACE inhibitory peptides are di- or tripeptides with the ability of binding to the buried active site of ACE [41]. Limited information is available in the literature that discusses correlation between the isoelectric point of peptides and their ACE inhibitory activity. Hydrophobicity values of identified bioactive dipeptides ranged from −0.05 to 0.43, while hydrophilicity values were in the range of −0.9 to 0.25. PL showed the highest hydrophobicity value (0.23) and lowest hydrophilicity value (−0.9). Therefore, the varying physicochemical parameters such as hydrophobicity and hydrophilicity indicate the potential of utilizing the predicted short-chain peptides derived from sea cucumber in a wide range of food systems, including water soluble, lipid soluble as well as in emulsions. In general, ACE inhibitory peptides are expected to exert their functionality in both hydrophilic and hydrophobic systems [42].

### 2.6. In Silico Analysis of Absorption, Distribution, Metabolism and Excretion (ADME) Profile of Bioactive Peptides Derived from Sea Cucumber Hydrolysates

The trend of using virtual screening for identifying the potential bioactive compounds has become an asset in drug discovery process [43]. Drug development assessments generally include absorption, distribution, and excretion (ADME) parameters. The web-based tool SwissADME provides robust predictive models and primary information for individual parameters important for drug development process [44]. Evaluation of ADME parameters at the initial stage mitigates the pharmacokinetics (i.e., the fate of a therapeutic compound in the organism) related failures in the clinical phase. SwissADME evaluation methods include the analysis of drug-likeness which investigates the probability to be an oral drug [44]. One of the most common and convenient delivery methods of peptide therapeutics is oral administration [45]. The selected criteria in the present study focused on the crucial physicochemical parameters used for drug designing, including number of rotatable bonds (ROTB), number of hydrogen bond acceptors, number of hydrogen bond donors and water solubility. Furthermore, polarity of the molecule was evaluated using topological polar surface area (TPSA) technique considering sulfur and phosphorus as polar atoms. TPSA is one of the key parameters to estimate the ADME properties [44]. Lipophilicity was analyzed based on the partition coefficient between octanol and water (log Po/w) and it has a vital impact on pharmacokinetics in drug discovery process. Bioavailability score and Lipinski filter were evaluated to assess the qualitative chance for sea cucumber derived peptides to become oral drugs with emphasis on bioavailability and structural characteristics. Lipinski filter is developed on the Lipinski (Pfizer’s) rule of five which is known as the rule of thumb to determine the drug-likeness of a molecule [42]. The main criteria of the rule include less than five hydrogen bond donors (the total number of nitrogen-hydrogen and oxygen-hydrogen bonds), no more than 10 hydrogen bond acceptors (all nitrogen or oxygen atoms), molecular mass of less than 500 Da, and an octanol-water partition coefficient (log P) greater than or equal to five. In general, orally active drugs can have only one exemption/violation of these criteria. All these parameters were evaluated on par with the standard captopril. As shown in Table 10, four of the five predicted dipeptides from sea cucumber protein hydrolysates were in accordance with all Lipinski criteria.

**Figure 1:**
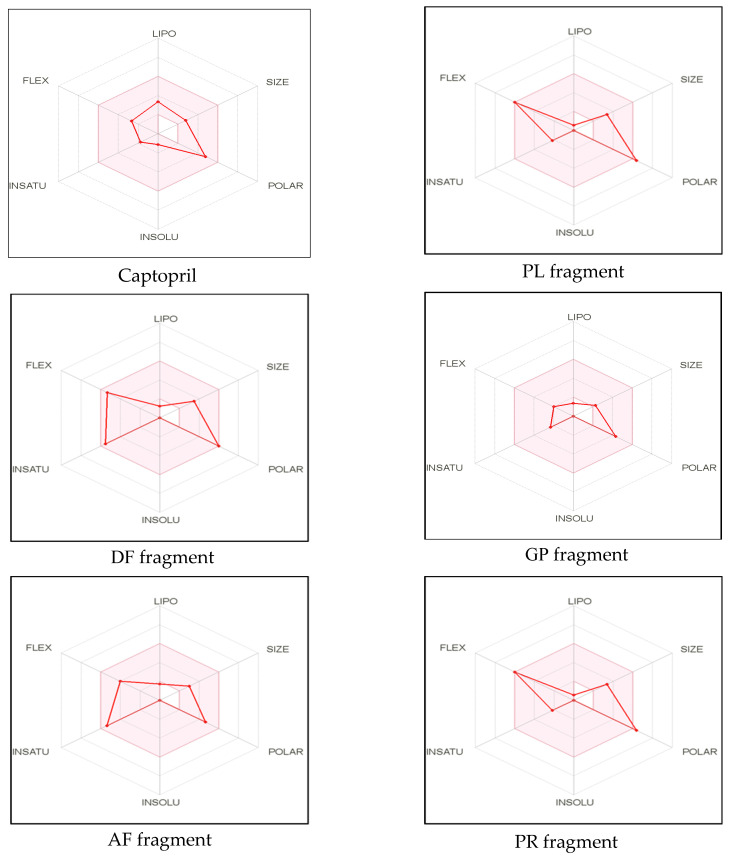
Bioavailability radar of sea cucumber-derived bioactive peptide and ACE inhibitory drug (captopril) based on physicochemical indices ideal for oral bioavailability.

The remaining dipeptide PR was also in agreement with Lipinski filter criteria with one violation (exceeded the maximum number of hydrogen bond donors (HBD)). Moreover, it has been suggested that compounds with more than 10 rotatable bonds show poor oral bioavailability whereas TPSA value ranging from 20 to 130Å^2^ are suitable for providing high oral bioavailability [42]. All peptides, except PR, exhibited low TPSA values and high gastrointestinal absorbance ability. Lipophilicity and polarity of compounds are crucial to determine the passive gastrointestinal digestion [46]. Furthermore, bioavailability scores of all the identified peptides were similar to that of standard inhibitory drug captopril. Bioavailability radar, a graphical representation of most of the above-mentioned parameters with some additional properties simplifies the evaluation of drug-likeness of a molecule. Six physicochemical properties, including lipophilicity, molecular weight/size, polarity, solubility, saturation, and flexibility of the molecule were displayed in the radar (Figure 1).

LIPO, Lipophilicity: −0.7 < XLOGP3 < +5.0; SIZE, Molecular size: 150 g/mol < mol. wt. < 500 g/mol; POLAR, Polarity: 20 Å^2^ < TPSA < 130 Å^2^; INSOLU, Insolubility: 0 < Log S (ESOL) < 6; INSATU, Instauration: 0.25 < Fraction Csp^3^ < 1; FLEX, Flexibility: 0 < Number of rotatable bonds < 9. The colored zone is the suitable physicochemical space for oral bioavailability

The pink area in the radar represents the favorable range for oral bioavailability which is based on the optimal values for each property. For example, lipophilicity which is indicated by the partition coefficient range between n-octanol and water (log Po/w), should range from −0.7 to +5.0. The molecular weight should be within the size of 150–500 Da and optimal range of TPSA should fall into 20–130 Å^2^. In addition, solubility which is denoted using the descriptor decimal logarithm of the molar solubility in water should not be higher than 6, whereas the fraction of carbons in the sp^3^ hybridization should not exceed 0.25. The optimum flexibility is accountable for having less than 9 rotatable bonds. However, the crucial determinants related to oral bioavailability of the molecule are flexibility and polarity [42,46]. In our study, except PR, all other dipeptides fall into the optimal range of each parameter (Figure 1). These findings suggest that identified bioactive dipeptides, excluding PR, have excellent conditions for oral bioavailability and better absorption ability in the GI tract. Thus, SwissADME classifiers predictions demonstrate that peptides derived from sea cucumber hydrolysates may possess drug-like properties, similar to standard inhibitory drugs (captopril) and have the potential to be used in the pharmaceutical industry. However, further experimental validation is needed for confirmation of the predicted properties.

## 3. Materials and Methods

### 3.1. Materials

Fresh sea cucumbers (*Cucumaria frondosa*) were harvested from Northwest and Southeast regions of the St. Pierre Bank (NAFO Division 3Ps), Newfoundland, Canada. Alcalase (2.4 AU/g) and Flavourzyme (1000 LAPU/g) were procured from Novozymes, Bagsvaerd, Denmark. All other chemicals used in the experiments were purchased from Sigma-Aldrich Canada Ltd. (Oakville, ON, Canada).

### 3.2. Preparation of Sea Cucumber Protein Hydrolysates

Protein isolation from sea cucumber by-products (i.e., flower and internal organs) was prepared based on isoelectric protein precipitation reported previously [11]. The isolated protein fractions were separately collected and lyophilized prior to hydrolysis. The protein hydrolysates were then prepared according to the method described by Ambigaipalan and Shahidi [7]. The extracted protein isolates from flower (FL) and internal organs (IN) of Atlantic Sea cucumbers were hydrolyzed by sequential addition of Alcalase (AL, 0.3 AU/g) and Flavourzyme (F, 50 LAPU/g) based on the findings of Senadheera et al. [11]. These enzymes are very common in industrial production due to their favorable operational conditions. Upon completion of the incubation period, reactions were terminated by heating the mixture at 90 °C for 10 min to inactivate the enzyme and samples was then freeze-dried until the time of analysis.

### 3.3. LC-MS/MS Analysis

LC-MS/MS analysis was carried out at the Analytics, Robotics and Chemical Biology Centre (SPARC BioCentre), the Hospital for Sick Children, Toronto, ON, Canada using—Exactive Orbitrap analyzer outfitted with a nanospray source and EASY-nLC nano-LC system (Thermo Fisher, San Jose, CA, USA), as reported by Udenigwe et al. [47] PEAKS X+ software (Bioinformatic Solutions, Waterloo, ON, Canada) was used to perform the data analysis.

### 3.4. In Silico Prediction of Bioactive Potential of Identified Peptides from Protein Hydrolysate Samples

Peptides identified using PEAKS X+ software were further analyzed for their bioactive potential using in silico tools. Peptides were screened according to their bioactive potential using PepRank (http://bioware.ucd.ie/~compass/biowareweb/ (accessed on 27 September 2020)), which predicts the probability (between 0 and 1) of the peptide being bioactive, as described by Mooney et al. [17]. The threshold of 0.9 was selected to reduce the number of false positives. Selected peptides were analyzed for their bioactive properties related to antioxidant and antihypertensive activities using BIOPEP-UWM^TM^ database (http://www.uwm.edu.pl/biochemia/index.php/en/biopep (accessed on 27 September 2020)) of bioactive peptides. The identified sequences with ACE inhibitory peptides were subjected to in silico hydrolysis using the enzyme(s) action feature of BIOPEP-UWM^TM^ by employing pepsin (EC 3.4.23.1), trypsin (EC 3.4.21.4) and chymotrypsin (EC 3.4.21.1) as representative digestive enzymes as described by Ji et al. [42].

### 3.5. Toxicity and Physicochemical Properties of Bioactive Peptides Released after in Silico Proteolysis

The potential toxicity, hydrophobicity, hydrophilicity, charge, isoelectric point and molecular weight of the peptides released after simulated digestive proteolysis were predicted using ToxinPred (https://webs.iiitd.edu.in/raghava/toxinpred/index.html (accessed on 2 October 2020)) web-based application according to the method described by Gupta et al. [38].

### 3.6. In Silico Analysis of Absorption, Distribution, Metabolism and Excretion (ADME) Profile of Bioactive Peptides

In silico analysis of drug-likeliness for identified sea cucumber derived-peptides was evaluated based on absorption, distribution, metabolism and excretion parameters using SwissADME tool (http://www.swissadme.ch/index.php# (accessed on 20 October 2020)) as explained by Daina et al. [44].

## 4. Conclusions

Based on the above, findings of this study indicate that bioinformatic approach can effectively evaluate the bioactive potential and physicochemical properties of peptides encrypted in sea cucumber by-products proteome. The current findings also provide an important consideration and a direction that can lead to utilize the undervalued sea cucumber by-products as precursor materials to generate oral therapeutic ingredients. Thus, the results of in silico analysis demonstrated that sea cucumber derived peptides present great potential for the development of nutraceutical products. However, the virtual predictions and simulations should be validated using structure-functional analysis and in vivo approaches for confirmation of bioactive properties.

## Figures and Tables

**Table 1:** Bioactive potential of identified peptides according to PEPRANK.

Sample	Total Identified Peptides over 0.9 Threshold	PepRank	Peptide Sequence
Flower	8	0.96	GPPGPQWPLDF
		0.94	GPPGPRGPTGRMG
		0.94	GPGGPGPGM
		0.93	APDMAFPR
		0.92	GGFPGGPG
		0.92	GPGMMGP
		0.90	GPPGASGPLGIAGSM
		0.90	GPSGPPGP
Internal organs	6	0.96	GPPGPQWPLDF
		0.94	GEPFPKF
		0.93	APDMAFPR
		0.92	PGGPGPGM
		0.92	GPGMMGP
		0.90	DPIFFPS

G—Glycine, P—Proline, Q—Glutamine, W—Tryptophan, L—Leucine, D—Aspartic acid, F—Phenylalanine, T—Threonine, M—Methionine, A—Alaninie, R—Arginine, S—Serine, I—Isoleucine, K—Lysine.

**Table 2:** In silico predictions of potential antioxidative peptides from flower protein hydrolysates.

Sample	Peptide Sequence	Name of Peptide	Activity	Bioactive Sequence	Location of Fragmentation
Flower	GPPGPQWPLDF	peptide from buckwheat	antioxidative	WPL	(7–9)
		Antioxidative peptide	antioxidative	GPP	(1–3)
	GPPGPRGPTGRMG	Antioxidative peptide	antioxidative	GPP	(1–3)
	GPGGPGPGM	_	_	_	_
	APDMAFPR	_	_	_	_
	GGFPGGPG	_	_	_	_
	GPGMMGP	Antioxidative peptide	antioxidative	MM	(5–6)
	GPPGASGPLGIAGSM	Antioxidative peptide	antioxidative	GPP	(1–3)
	GPSGPPGP	Antioxidative peptide	antioxidative	GPP	(4–6)

G—Glycine, P—Proline, Q—Glutamine, W—Tryptophan, L—Leucine, D—Aspartic acid, F—Phenylalanine, T—Threonine, M—Methionine, A—Alanine, R—Arginine, S—Serine, I—Isoleucine, K—Lysine.

**Table 3:** In silico predictions of potential antioxidative peptides from internal organs hydrolysates.

Sample	Peptide Sequence	Name of Peptide	Activity	Bioactive Sequence	Location of Fragmentation
Internal organs	GPPGPQWPLDF	Peptide from buckwheat	antioxidative	WPL	(7–9)
		Antioxidative peptide	antioxidative	GPP	(1–3)
	GEPFPKF	_	_	_	_
	APDMAFPR	_	_	_	_
	PGGPGPGM	_	_	_	_
	GPGMMGP	Antioxidative peptide	antioxidative	MM	(5–6)
	DPIFFPS	_	_	_	_

G—Glycine, P—Proline, Q—Glutamine, W—Tryptophan, L—Leucine, D—Aspartic acid, E—Glutamic acid, F—Phenylalanine, T—Threonine, M—Methionine, A—Alanine, R—Arginine, S—Serine, I—Isoleucine, K—Lysine.

**Table 4:** In silico predictions of potential ACE inhibitory active peptides from flower protein hydrolysates.

Sample	Peptide Sequence	Name of Peptide Appears in the Data Base	Bioactive Sequence	Location of Fragmentation
Flower	GPPGPQWPLDF	ACE inhibitor from Alaskan pollack skin	GP	(1–2), (4–5)
		ACE inhibitor from Alaskan pollack skin	PL	(8–9)
		ACE inhibitor	PG	(3–4)
		ACE inhibitor from wheat gliadin	GPP	(1–3)
		ACE inhibitor	PP	(2–3)
		ACE inhibitor	PQ	(5–6)
		ACE inhibitor	DF	(10–11)
	GPPGPRGPTGRMG	ACE inhibitor	PR	(5–6)
		ACE inhibitor from Alaskan pollack skin	GP	(1–2), (4–5), (7–8)
		ACE inhibitor	GR	(10–11)
		ACE inhibitor	MG	(12–13)
		ACE inhibitor	TG	(9–10)
		ACE inhibitor	PG	(3–4)
		ACE inhibitor from wheat gliadin	GPP	(1–3)
		ACE inhibitor	PT	(8–9)
		ACE inhibitor	PP	(2–3)
		ACE inhibitor	RG	(6–7)
	GPGGPGPGM	ACE inhibitor from Alaskan pollack skin	GP	(1–2), (4–5), (6–7)
		ACE inhibitor	GM	(8–9)
		ACE inhibitor	GG	(3–4)
		ACE inhibitor	PG	(2–3), (5–6), (7–8)
	APDMAFPR	ACE inhibitor	FP	(6–7)
		ACE inhibitor	PR	(7–8)
			AFP	(5–7)
		ACE inhibitor	AF	(5–6)
		ACE inhibitor	AP	(1–2)
		ACE inhibitor	DM	(3–4)
	GGFPGGPG	ACE inhibitor	FP	(4–5)
		ACE inhibitor from Alaskan pollack skin	GP	(7–8)
		ACE inhibitor	GF	(3–4)
		ACE inhibitor	GG	(2–3), (6–7)
		ACE inhibitor	PG	(5–6), (8–9)
	GPGMMGP	ACE inhibitor	GM	(4–5)
		ACE inhibitor	MG	(6–7)
		ACE inhibitor	PG	(3–4)
		ACE inhibitor	MM	(5–6)
		ACE inhibitor	MGP	(6–8)
	GPPGASGPLGIAGSM	ACE inhibitor from Alaskan pollack skin	GPL	(7–9)
		ACE inhibitor from Alaskan pollack skin	PLG	(8–10)
		ACE inhibitor from Alaskan pollack skin	GP	(1–2), (7–8)
		ACE inhibitor from Alaskan pollack skin	PL	(8–9)
		ACE inhibitor from soy hydrolysate	IA	(11–12)
		ACE inhibitor	GI	(10–11)
		ACE inhibitor	GA	(4–5)
		ACE inhibitor	AG	(12–13)
		ACE inhibitor	GS	(13–14)
		ACE inhibitor	SG	(6–7)
		ACE inhibitor	LG	(9–10)
		ACE inhibitor	PG	(3–4)
		ACE inhibitor from wheat gliadin	GPP	(1–3)
		ACE inhibitor	PP	(2–3)
		ACE inhibitor	LGI	(9–11)
		ACE inhibitor	SGP	(6–8)
		ACE inhibitor	AGS	(12–14)
	GPSGPPGP	ACE inhibitor from Alaskan pollack skin	GP	(1–2), (4–5), (7–8)
		ACE inhibitor	SG	(3–4)
		ACE inhibitor	PG	(6–7)
		ACE inhibitor from wheat gliadin	GPP	(4–6)
		ACE inhibitor	PP	(5–6)
		ACE inhibitor	SGP	(3–5)

G—Glycine, P—Proline, Q—Glutamine, W—Tryptophan, L—Leucine, D—Aspartic acid, F—Phenylalanine, T—Threonine, M—Methionine, A—Alanine, R—Arginine, S—Serine, I—Isoleucine, K—Lysine.

**Table 5:** In silico predictions of potential ACE inhibitory active peptides from internal organs hydrolysates.

Sample	Peptide Sequence	Name of Peptide	Bioactive Sequence	Location of Fragmentation
Internal organs	GPPGPQWPLDF	ACE inhibitor from Alaskan pollack skin	GP	(1–2), (4–5)
		ACE inhibitor from Alaskan pollack skin	PL	(8–9)
		ACE inhibitor	PG	(3–4)
		ACE inhibitor from wheat gliadin	GPP	(1–3)
		ACE inhibitor	PP	(2–3)
		ACE inhibitor	PQ	(5–6)
		ACE inhibitor	DF	(10–11)
	GEPFPKF	ACE inhibitor	FP	(4–5)
		ACE inhibitor from Tricholoma giganteum	GEP	(1–3)
		ACE inhibitor	GE	(1–2)
		ACE inhibitor	KF	(6–7)
		ACE inhibitor	PFP	(3–5)
	APDMAFPR	ACE inhibitor	FP	(7–8)
		ACE inhibitor	PR	(8–9)
			AFP	(6–8)
		ACE inhibitor	AF	(6–7)
		ACE inhibitor	AP	(2–3)
		ACE inhibitor	DM	(4–5)
	PGGPGPGM	ACE inhibitor from Alaskan pollack skin	GP	(3–4), (5–6)
		ACE inhibitor	GM	(7–8)
		ACE inhibitor	GG	(2–3)
		ACE inhibitor	PG	(1–2), (4–5), (6–7)
	GPGMMGP	ACE inhibitor from Alaskan pollack skin	GP	(2–3), (7–8)
		ACE inhibitor	GM	(4–5)
		ACE inhibitor	MG	(6–7)
		ACE inhibitor	PG	(3–4)
		ACE inhibitor	MM	(5–6)
		ACE inhibitor	MGP	(6–8)
	DPIFFPS	ACE inhibitor	FP	(5–6)
		ACE inhibitor	IF	(3–4)

G—Glycine, P—Proline, Q—Glutamine, W—Tryptophan, L—Leucine, D—Aspartic acid, F—Phenylalanine, T—Threonine, M—Methionine, A—Alanine, R—Arginine, S—Serine, I—Isoleucine, K—Lysine.

**Table 6:** Remaining bioactive properties after in silico simulated gastrointestinal digestion of peptides originated from flower protein hydrolysates.

Sample	Peptide	Results of Enzyme Action	Location of Released Peptides	Active Fragment Sequence	Location	Bioactivity of Identified Peptide
Flower	GPPGPQWPLDF	GPPGPQW—PL—DF	(1–7), (8–9), (10–11)	PL	(8–9)	ACE inhibitor
						
				DF	(10–11)	ACE inhibitor
	GPPGPRGPTGRMG	GPPGPR—GPTGR—M—G	(1–6), (7–11), (12–12), (13–13)	_	_	No Antioxidant or ACE inhibitory activity
						
	GPGGPGPGM	_	_	_	_	_
						
	APDMAFPR	APDM—AF—PR	(1–4), (5–6), (7–8)	PR	(7–8)	ACE inhibitor
				AF	(5–6)	ACE inhibitor
	GGFPGGPG	GGF—PGGPG	(1–3), (4–8)	_	_	No Antioxidant or ACE inhibitory activity
						
	GPGMMGP	GPGM—M—GP	(1–4), (5–5), (6–7)	GP	(6–7)	ACE inhibitor
						
	GPPGASGPLGIAGSM	GPPGASGPL—GIAGSM	(1–9), (10–15)	_	_	No Antioxidant or ACE inhibitory activity
						
	GPSGPPGP	_	_	_	_	_

G—Glycine, P—Proline, Q—Glutamine, W—Tryptophan, L—Leucine, D—Aspartic acid, F—Phenylalanine, T—Threonine, M—Methionine, A—Alanine, R—Arginine, S—Serine, I—Isoleucine, K—Lysine.

**Table 7:** Remaining Antioxidant or ACE inhibitory properties after in silico simulated gastrointestinal digestion of peptides originated from internal organs protein hydrolysates.

Sample	Peptide	Results of Enzyme Action	Location of Released Peptides	Active Fragment Sequence	Location	Bioactivity of Identified Peptide
Internal organs	GPPGPQWPLDF	GPPGPQW—PL—DF	(1–7), (8–9), (10–11)	PL	(8–9)	ACE inhibitor
				DF	(10–11)	ACE inhibitor
	GEPFPKF	GEPF—PK—F	(1–4), (5–6), (7–7)	_	_	No Antioxidant or ACE inhibitory activity
						
	APDMAFPR	APDM—AF—PR	(1–4), (5–6), (7–8)	PR	(7–8)	ACE inhibitor
				AF	(5–6)	ACE inhibitor
						
	PGGPGPGM	_	_	_	_	_
						
	GPGMMGP	GPGM—M—GP	(1–4), (5–5), (6–7)	GP	(6–7)	ACE inhibitor
						
	DPIFFPS	DPIF—F—PS	(1–4), (5–5), (6–7)	_	_	No Antioxidant or ACE inhibitory activity

G—Glycine, P—Proline, Q—Glutamine, W—Tryptophan, L—Leucine, D—Aspartic acid, F—Phenylalanine, T—Threonine, M—Methionine, A—Alanine, R—Arginine, S—Serine, I—Isoleucine, K—Lysine.

**Table 8:** In silico hydrolysis performance and physicochemical characteristics of bioactive peptides remaining after simulated in silico digestion.

Peptide	Sample	Active Fragment Sequence	Location	DHt (%)	A_E_	W
GPPGPQWPLDF	flower, internal organs	PL DF	(8–9) (10–1)	20	0.18	0.25
GPGMMGP	Flower internal organs	GP	(6–7)	33.33	0.14	0.14
APDMAFPR	flower, internal organs	AF PR	(5–6) (7–8)	28.57	0.25	0.33

G—Glycine, P—Proline, Q—Glutamine, W—Tryptophan, L—Leucine, D—Aspartic acid, F—Phenylalanine, M—Methionine, A—Alanine, R—Arginine.

**Table 9:** Physicochemical properties and toxicity of bioactive fragments releasing after the simulation in silico digestion.

Active Fragment Sequence	Prediction	Hydrophobicity	Hydrophilicity	Charge	pI	Molecular Weight (Da)
PL	Non-toxic	0.23	−0.9	0	5.88	228.31
DF	Non-toxic	−0.05	0.25	−1	3.8	280.29
GP	Non-toxic	0.04	0	0	5.88	172.20
AF	Non-toxic	0.43	−1.5	0	5.88	236.28
PR	Non-toxic	−0.92	1.5	1	10.11	271.33

G—Glycine, P—Proline, L—Leucine, D—Aspartic acid, F—Phenylalanine, A—Alanine, R—Arginine.

**Table 10:** In silico absorption, distribution, metabolism, excretion (ADME), and physicochemical properties of bioactive peptides.

Active Fragment Sequence	Physicochemical Properties				Lipophilicity		Drug Likeliness		Pharmacokinetics
	ROTB	HBA	HBD	ESOL	TPSA (Å^2^)	C LogP	Bioavailability Score	Lipinski Filter	GIA
Captopril	4	3	1	−1.14 Very soluble	96.41	0.62	0.56	Yes (0)	High
PL	6	4	3	0.66 Highly soluble	78.43	0.04	0.55	Yes (0)	High
DF	8	6	4	0.83 Highly soluble	129.72	−0.69	0.56	Yes (0)	High
GP	3	4	2	1.25 Highly soluble	83.63	−1.17	0.55	Yes (0)	High
AF	6	4	3	0.39 Highly soluble	92.42	0.05	0.55	Yes (0)	High
PR	9	5	6	1.94 Highly soluble	140.33	−1.59	0.55	Yes (1)	Low

G—Glycine, P—Proline, L—Leucine, D—Aspartic acid, F—Phenylalanine, A—Alanine, R—Arginine.
